# (2,4-Dimethoxy­benzyl­idene)-2-hydroxy­benzohydrazide ethanol solvate

**DOI:** 10.1107/S1600536808011768

**Published:** 2008-05-03

**Authors:** Wagee A. Yehye, Azhar Ariffin, Seik Weng Ng

**Affiliations:** a Department of Chemistry, University of Malaya, 50603 Kuala Lumpur, Malaysia

## Abstract

In the planar title mol­ecule, C_16_H_16_N_2_O_4_·C_2_H_6_O, the planar Schiff base molecule is linked to the ethanol solvent mol­ecule by a hydr­oxy–amide hydrogen bond. The hydr­oxy group of the ethanol mol­ecule is a hydrogen-bond donor to the double-bonded N atom of an adjacent Sciff base, pairs of interactions taking place across a center of symmetry and giving rise to a hydrogen-bonded dimer.

## Related literature

For the crystal structures of other substituted benzyl­idene-2-hydroxy­benzohydrazides, see: Li (2007 ▶); Liang *et al.* (2005 ▶); Luo (2007 ▶); Ma *et al.* (2005 ▶); Pan & Yang (2005*a* ▶,*b* ▶,*c* ▶); Qiu *et al.* (2006 ▶); Shao *et al.* (2004 ▶); Wang *et al.* (2007 ▶); Xu & Liu (2006 ▶); Yang (2006 ▶); Yang & Pan (2004 ▶, 2005*a* ▶,*b* ▶); Zhang *et al.* (2006 ▶).
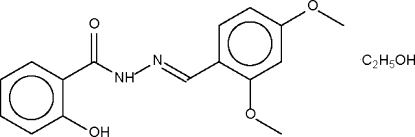


## Experimental

### 

#### Crystal data

C_16_H_16_N_2_O_4_·C_2_H_6_O
*M* *_r_* = 346.38
Monoclinic, 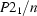

*a* = 7.7909 (2) Å
*b* = 18.0539 (6) Å
*c* = 12.0001 (4) Å
β = 93.803 (2)°
*V* = 1684.17 (9) Å^3^
*Z* = 4
Mo *K*α radiation
μ = 0.10 mm^−1^
*T* = 100 (2) K
0.20 × 0.15 × 0.15 mm

#### Data collection

Bruker SMART APEX diffractometer
Absorption correction: none
13796 measured reflections
3853 independent reflections
2575 reflections with *I* > 2σ(*I*)
*R*_int_ = 0.059

#### Refinement

*R*[*F* ^2^ > 2σ(*F* ^2^)] = 0.046
*wR*(*F* ^2^) = 0.118
*S* = 1.03
3853 reflections
241 parameters
3 restraints
H atoms treated by a mixture of independent and constrained refinement
Δρ_max_ = 0.21 e Å^−3^
Δρ_min_ = −0.24 e Å^−3^

### 

Data collection: *APEX2* (Bruker, 2007 ▶); cell refinement: *SAINT* (Bruker, 2007 ▶); data reduction: *SAINT*; program(s) used to solve structure: *SHELXS97* (Sheldrick, 2008 ▶); program(s) used to refine structure: *SHELXL97* (Sheldrick, 2008 ▶); molecular graphics: *X-SEED* (Barbour, 2001 ▶); software used to prepare material for publication: *publCIF* (Westrip, 2008 ▶).

## Supplementary Material

Crystal structure: contains datablocks global, I. DOI: 10.1107/S1600536808011768/sg2239sup1.cif

Structure factors: contains datablocks I. DOI: 10.1107/S1600536808011768/sg2239Isup2.hkl

Additional supplementary materials:  crystallographic information; 3D view; checkCIF report

## Figures and Tables

**Table 1:** Hydrogen-bond geometry (Å, °)

*D*—H⋯*A*	*D*—H	H⋯*A*	*D*⋯*A*	*D*—H⋯*A*
O1—H1o⋯O2	0.86 (1)	1.74 (2)	2.528 (2)	151 (2)
O5—H5o⋯N2^i^	0.85 (1)	2.07 (1)	2.847 (2)	152 (2)
N1—H1n⋯O5	0.86 (1)	2.09 (1)	2.894 (2)	157 (2)

Symmetry code: (i) 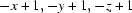.

**Fig. 1.**
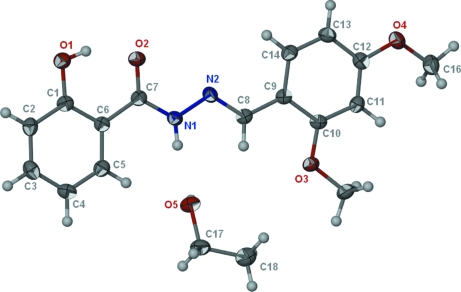
Thermal ellipsoid plot (Barbour, 2001) of C16H16N2O4.C2H6O at the 70% probability level. Hydrogen atoms are drawn as spheres of arbitrary radiius.

**Fig. 2.**
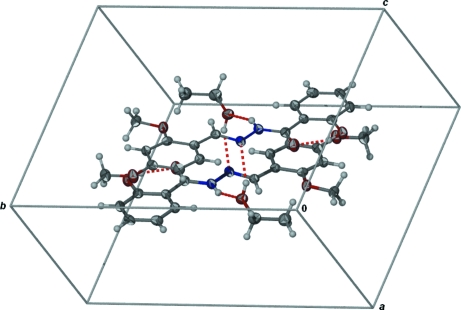
Hydrogen-bonded dimeric structure.

## supplementary crystallographic information

### Comment

The crystal structures of a number of substituted benzylidene-2-hydroxybenzohydrazides have been reported (Li, 2007; Liang *et al.*, 2005; Luo, 2007; Ma *et al.*, 2005; Pan & Yang, 2005*a*,*b*,*c*; Qiu *et al.*, 2006; Shao *et al.*, 2004; Wang *et al.*, 2007; Xu & Liu, 2006; Yang, 2006; Yang & Pan, 2004, 2005*a*,*b*; Zhang *et al.*, 2006.

The 2,4-dimethoxy derivative crystallizes as an ethanol solvate (Scheme I, Fig. 1). The planar molecule of C_16_H_16_N_2_O_4_ is linked to the ethanol molecule by an amido···hydroxy_ethanol_ hydrogen bond [N–H···O 2.894 (2) Å]. The hydroxy unit of the ethanol molecule is a hydrogen-bond donor site to the double-bond nitrogen atom of an adjacent Sciff base [O–H···N 2.847 (2) Å], this interaction across a center of symmetry giving rise to a hydrogen-bonded dimer (Fig. 2).

### Experimental

2-Hydroxybenzohydrazide (0.60 g, 4 mmol) and 2,4-dimethoxybenzaldehyde (0.66 g, 4 mmol) were heated in ethanol (30 ml) for 2 h. The solvent was removed by evaporation and the product recrystallized from ethanol.

### Refinement

Carbon-bound H-atoms were placed in calculated positions (C—H 0.95 to 0.99 Å) and were included in the refinement in the riding model approximation, with *U*(H) set to 1.2–1.5 *U*(C).

The oxygen- and nitrogen-bound H-atoms were located in a difference Fouier map, and were refined with a distance restraint (O–H = N–H 0.85 Å); their temperature factors were freely refined.

### Crystal data

**Table:**

C_16_H_16_N_2_O_4_·C_2_H_6_O	*F*_000_ = 736
*M**_r_* = 346.38	*D*_x_ = 1.366 Mg m^−^^3^
Monoclinic, *P*2_1_/*n*	Mo *K*α radiation λ = 0.71073 Å
Hall symbol: -P 2yn	Cell parameters from 1895 reflections
*a* = 7.7909 (2) Å	θ = 2.8–25.5º
*b* = 18.0539 (6) Å	µ = 0.10 mm^−^^1^
*c* = 12.0001 (4) Å	*T* = 100 (2) K
β = 93.803 (2)º	Prism, colorless
*V* = 1684.17 (9) Å^3^	0.20 × 0.15 × 0.15 mm
*Z* = 4	

### Data collection

**Table:**

Bruker SMART APEX diffractometer	2575 reflections with *I* > 2σ(*I*)
Radiation source: fine-focus sealed tube	*R*_int_ = 0.059
Monochromator: graphite	θ_max_ = 27.5º
*T* = 100(2) K	θ_min_ = 2.0º
ω scans	*h* = −10→10
Absorption correction: None	*k* = −20→23
13796 measured reflections	*l* = −15→15
3853 independent reflections	

### Refinement

**Table:**

Refinement on *F*^2^	Secondary atom site location: difference Fourier map
Least-squares matrix: full	Hydrogen site location: inferred from neighbouring sites
*R*[*F*^2^ > 2σ(*F*^2^)] = 0.046	H atoms treated by a mixture of independent and constrained refinement
*wR*(*F*^2^) = 0.118	*w* = 1/[σ^2^(*F*_o_^2^) + (0.0476*P*)^2^ + 0.175*P*] where *P* = (*F*_o_^2^ + 2*F*_c_^2^)/3
*S* = 1.03	(Δ/σ)_max_ = 0.001
3853 reflections	Δρ_max_ = 0.21 e Å^−^^3^
241 parameters	Δρ_min_ = −0.24 e Å^−^^3^
3 restraints	Extinction correction: none
Primary atom site location: structure-invariant direct methods	

### Fractional atomic coordinates and isotropic or equivalent isotropic displacement parameters (Å^2^)

**Table:**

	*x*	*y*	*z*	*U*_iso_*/*U*_eq_	
O1	0.64682 (15)	0.22307 (7)	0.69448 (11)	0.0248 (3)	
O2	0.48542 (15)	0.30514 (7)	0.55256 (10)	0.0253 (3)	
O3	0.09164 (14)	0.62457 (7)	0.44328 (10)	0.0224 (3)	
O4	−0.15888 (16)	0.55131 (7)	0.07909 (10)	0.0280 (3)	
O5	0.45339 (16)	0.56902 (7)	0.68732 (11)	0.0256 (3)	
N1	0.40917 (17)	0.42200 (9)	0.59366 (12)	0.0191 (3)	
N2	0.32044 (17)	0.42928 (8)	0.48948 (11)	0.0195 (3)	
C1	0.6546 (2)	0.28297 (10)	0.76338 (14)	0.0199 (4)	
C2	0.7381 (2)	0.27412 (10)	0.86870 (14)	0.0219 (4)	
H2	0.7858	0.2275	0.8904	0.026*	
C3	0.7514 (2)	0.33316 (11)	0.94120 (14)	0.0231 (4)	
H3	0.8073	0.3266	1.0133	0.028*	
C4	0.6847 (2)	0.40220 (10)	0.91101 (14)	0.0230 (4)	
H4	0.6962	0.4428	0.9614	0.028*	
C5	0.6014 (2)	0.41094 (10)	0.80671 (14)	0.0211 (4)	
H5	0.5560	0.4581	0.7856	0.025*	
C6	0.5825 (2)	0.35172 (10)	0.73132 (14)	0.0180 (4)	
C7	0.4908 (2)	0.35796 (10)	0.61929 (14)	0.0192 (4)	
C8	0.22902 (19)	0.48872 (10)	0.47864 (14)	0.0188 (4)	
H8	0.2277	0.5226	0.5391	0.023*	
C9	0.1282 (2)	0.50446 (10)	0.37526 (14)	0.0185 (4)	
C10	0.0552 (2)	0.57510 (10)	0.35888 (14)	0.0189 (4)	
C11	−0.0433 (2)	0.59274 (10)	0.26156 (14)	0.0197 (4)	
H11	−0.0930	0.6405	0.2519	0.024*	
C12	−0.0677 (2)	0.53942 (10)	0.17899 (14)	0.0213 (4)	
C13	0.0016 (2)	0.46857 (10)	0.19353 (15)	0.0227 (4)	
H13	−0.0173	0.4321	0.1369	0.027*	
C14	0.0973 (2)	0.45200 (10)	0.29057 (14)	0.0205 (4)	
H14	0.1438	0.4036	0.3004	0.025*	
C15	0.0125 (2)	0.69592 (10)	0.43449 (15)	0.0240 (4)	
H15A	0.0456	0.7250	0.5015	0.036*	
H15B	−0.1128	0.6902	0.4275	0.036*	
H15C	0.0505	0.7215	0.3685	0.036*	
C16	−0.2167 (2)	0.62483 (11)	0.05390 (16)	0.0281 (4)	
H16A	−0.2707	0.6264	−0.0222	0.042*	
H16B	−0.1184	0.6588	0.0597	0.042*	
H16C	−0.3006	0.6398	0.1069	0.042*	
C17	0.4115 (2)	0.62589 (11)	0.76362 (15)	0.0278 (4)	
H17A	0.5186	0.6506	0.7929	0.033*	
H17B	0.3567	0.6033	0.8275	0.033*	
C18	0.2921 (2)	0.68255 (11)	0.70951 (16)	0.0310 (5)	
H18A	0.2608	0.7187	0.7655	0.046*	
H18B	0.1881	0.6579	0.6775	0.046*	
H18C	0.3496	0.7079	0.6502	0.046*	
H1O	0.594 (3)	0.2375 (13)	0.6331 (12)	0.054 (7)*	
H5O	0.518 (2)	0.5858 (13)	0.6389 (15)	0.052 (7)*	
H1N	0.410 (3)	0.4596 (8)	0.6371 (14)	0.036 (6)*	

### Atomic displacement parameters (Å^2^)

**Table:**

	*U*^11^	*U*^22^	*U*^33^	*U*^12^	*U*^13^	*U*^23^
O1	0.0311 (7)	0.0185 (7)	0.0241 (7)	0.0015 (5)	−0.0021 (6)	−0.0001 (6)
O2	0.0328 (7)	0.0185 (7)	0.0236 (7)	0.0024 (5)	−0.0045 (5)	−0.0035 (6)
O3	0.0291 (6)	0.0170 (7)	0.0208 (7)	0.0034 (5)	−0.0009 (5)	−0.0022 (5)
O4	0.0342 (7)	0.0261 (8)	0.0225 (7)	0.0032 (6)	−0.0077 (5)	0.0004 (6)
O5	0.0313 (7)	0.0212 (7)	0.0247 (7)	0.0003 (6)	0.0063 (6)	−0.0036 (6)
N1	0.0227 (7)	0.0181 (8)	0.0163 (8)	−0.0002 (6)	−0.0007 (6)	−0.0006 (7)
N2	0.0206 (7)	0.0205 (8)	0.0169 (8)	−0.0006 (6)	−0.0012 (6)	0.0010 (7)
C1	0.0190 (8)	0.0193 (10)	0.0218 (9)	−0.0023 (7)	0.0033 (7)	−0.0003 (8)
C2	0.0212 (8)	0.0210 (10)	0.0233 (10)	0.0009 (7)	0.0000 (7)	0.0044 (8)
C3	0.0224 (8)	0.0285 (11)	0.0183 (9)	−0.0043 (8)	0.0001 (7)	0.0050 (8)
C4	0.0263 (9)	0.0235 (10)	0.0194 (9)	−0.0042 (7)	0.0031 (7)	−0.0034 (8)
C5	0.0221 (8)	0.0181 (10)	0.0232 (10)	−0.0003 (7)	0.0022 (7)	0.0017 (8)
C6	0.0192 (8)	0.0169 (10)	0.0181 (9)	−0.0020 (7)	0.0018 (6)	0.0009 (7)
C7	0.0187 (8)	0.0176 (10)	0.0214 (9)	−0.0012 (7)	0.0024 (7)	−0.0001 (8)
C8	0.0190 (8)	0.0181 (9)	0.0198 (9)	−0.0010 (7)	0.0044 (7)	0.0001 (8)
C9	0.0191 (8)	0.0187 (9)	0.0179 (9)	−0.0014 (7)	0.0032 (6)	0.0005 (8)
C10	0.0181 (8)	0.0193 (10)	0.0196 (9)	−0.0026 (7)	0.0043 (7)	−0.0026 (8)
C11	0.0195 (8)	0.0179 (10)	0.0218 (9)	0.0012 (7)	0.0027 (7)	0.0028 (8)
C12	0.0210 (8)	0.0228 (10)	0.0200 (9)	−0.0026 (7)	0.0009 (7)	0.0018 (8)
C13	0.0253 (9)	0.0206 (10)	0.0218 (10)	−0.0024 (7)	−0.0006 (7)	−0.0034 (8)
C14	0.0217 (8)	0.0158 (9)	0.0240 (10)	−0.0014 (7)	0.0023 (7)	0.0007 (8)
C15	0.0268 (9)	0.0187 (10)	0.0267 (10)	0.0036 (7)	0.0032 (7)	−0.0032 (8)
C16	0.0288 (9)	0.0294 (11)	0.0254 (10)	0.0027 (8)	−0.0031 (8)	0.0047 (9)
C17	0.0319 (10)	0.0270 (11)	0.0245 (10)	0.0004 (8)	0.0026 (8)	−0.0066 (9)
C18	0.0323 (10)	0.0270 (12)	0.0339 (11)	0.0002 (8)	0.0040 (8)	−0.0023 (9)

### Geometric parameters (Å, °)

**Table:**

O1—C1	1.360 (2)	C8—C9	1.452 (2)
O1—H1O	0.859 (9)	C8—H8	0.9500
O2—C7	1.244 (2)	C9—C14	1.398 (2)
O3—C10	1.366 (2)	C9—C10	1.405 (2)
O3—C15	1.429 (2)	C10—C11	1.391 (2)
O4—C12	1.369 (2)	C11—C12	1.385 (2)
O4—C16	1.428 (2)	C11—H11	0.9500
O5—C17	1.428 (2)	C12—C13	1.395 (2)
O5—H5O	0.848 (10)	C13—C14	1.374 (2)
N1—C7	1.345 (2)	C13—H13	0.9500
N1—N2	1.3941 (19)	C14—H14	0.9500
N1—H1N	0.855 (9)	C15—H15A	0.9800
N2—C8	1.290 (2)	C15—H15B	0.9800
C1—C2	1.392 (2)	C15—H15C	0.9800
C1—C6	1.405 (2)	C16—H16A	0.9800
C2—C3	1.375 (2)	C16—H16B	0.9800
C2—H2	0.9500	C16—H16C	0.9800
C3—C4	1.389 (3)	C17—C18	1.501 (3)
C3—H3	0.9500	C17—H17A	0.9900
C4—C5	1.380 (2)	C17—H17B	0.9900
C4—H4	0.9500	C18—H18A	0.9800
C5—C6	1.402 (2)	C18—H18B	0.9800
C5—H5	0.9500	C18—H18C	0.9800
C6—C7	1.485 (2)		
			
C1—O1—H1O	106.2 (16)	C11—C10—C9	121.39 (16)
C10—O3—C15	117.86 (13)	C12—C11—C10	118.89 (16)
C12—O4—C16	117.97 (14)	C12—C11—H11	120.6
C17—O5—H5O	110.7 (17)	C10—C11—H11	120.6
C7—N1—N2	119.00 (15)	O4—C12—C11	123.77 (16)
C7—N1—H1N	123.9 (14)	O4—C12—C13	115.29 (16)
N2—N1—H1N	117.1 (14)	C11—C12—C13	120.94 (16)
C8—N2—N1	113.99 (14)	C14—C13—C12	119.31 (17)
O1—C1—C2	117.39 (16)	C14—C13—H13	120.3
O1—C1—C6	122.35 (15)	C12—C13—H13	120.3
C2—C1—C6	120.27 (16)	C13—C14—C9	121.74 (17)
C3—C2—C1	119.77 (17)	C13—C14—H14	119.1
C3—C2—H2	120.1	C9—C14—H14	119.1
C1—C2—H2	120.1	O3—C15—H15A	109.5
C2—C3—C4	121.28 (16)	O3—C15—H15B	109.5
C2—C3—H3	119.4	H15A—C15—H15B	109.5
C4—C3—H3	119.4	O3—C15—H15C	109.5
C5—C4—C3	118.97 (17)	H15A—C15—H15C	109.5
C5—C4—H4	120.5	H15B—C15—H15C	109.5
C3—C4—H4	120.5	O4—C16—H16A	109.5
C4—C5—C6	121.40 (17)	O4—C16—H16B	109.5
C4—C5—H5	119.3	H16A—C16—H16B	109.5
C6—C5—H5	119.3	O4—C16—H16C	109.5
C5—C6—C1	118.29 (15)	H16A—C16—H16C	109.5
C5—C6—C7	123.38 (16)	H16B—C16—H16C	109.5
C1—C6—C7	118.32 (15)	O5—C17—C18	111.97 (15)
O2—C7—N1	121.10 (16)	O5—C17—H17A	109.2
O2—C7—C6	121.22 (15)	C18—C17—H17A	109.2
N1—C7—C6	117.66 (15)	O5—C17—H17B	109.2
N2—C8—C9	120.86 (16)	C18—C17—H17B	109.2
N2—C8—H8	119.6	H17A—C17—H17B	107.9
C9—C8—H8	119.6	C17—C18—H18A	109.5
C14—C9—C10	117.70 (15)	C17—C18—H18B	109.5
C14—C9—C8	123.06 (16)	H18A—C18—H18B	109.5
C10—C9—C8	119.23 (16)	C17—C18—H18C	109.5
O3—C10—C11	123.27 (16)	H18A—C18—H18C	109.5
O3—C10—C9	115.32 (15)	H18B—C18—H18C	109.5
			
C7—N1—N2—C8	171.60 (15)	N2—C8—C9—C14	12.2 (2)
O1—C1—C2—C3	179.24 (15)	N2—C8—C9—C10	−168.70 (15)
C6—C1—C2—C3	−0.6 (2)	C15—O3—C10—C11	5.3 (2)
C1—C2—C3—C4	−0.8 (2)	C15—O3—C10—C9	−176.25 (14)
C2—C3—C4—C5	1.0 (2)	C14—C9—C10—O3	−178.97 (14)
C3—C4—C5—C6	0.2 (2)	C8—C9—C10—O3	1.9 (2)
C4—C5—C6—C1	−1.6 (2)	C14—C9—C10—C11	−0.5 (2)
C4—C5—C6—C7	178.61 (15)	C8—C9—C10—C11	−179.61 (15)
O1—C1—C6—C5	−178.08 (14)	O3—C10—C11—C12	177.63 (14)
C2—C1—C6—C5	1.7 (2)	C9—C10—C11—C12	−0.7 (2)
O1—C1—C6—C7	1.8 (2)	C16—O4—C12—C11	6.8 (2)
C2—C1—C6—C7	−178.44 (14)	C16—O4—C12—C13	−173.16 (15)
N2—N1—C7—O2	−1.3 (2)	C10—C11—C12—O4	−178.49 (15)
N2—N1—C7—C6	−179.38 (13)	C10—C11—C12—C13	1.5 (2)
C5—C6—C7—O2	175.88 (16)	O4—C12—C13—C14	179.01 (14)
C1—C6—C7—O2	−4.0 (2)	C11—C12—C13—C14	−0.9 (3)
C5—C6—C7—N1	−6.1 (2)	C12—C13—C14—C9	−0.3 (3)
C1—C6—C7—N1	174.10 (15)	C10—C9—C14—C13	1.0 (2)
N1—N2—C8—C9	−179.75 (14)	C8—C9—C14—C13	−179.89 (15)

### Hydrogen-bond geometry (Å, °)

**Table:**

*D*—H···*A*	*D*—H	H···*A*	*D*···*A*	*D*—H···*A*
O1—H1o···O2	0.86 (1)	1.74 (2)	2.528 (2)	151 (2)
O5—H5o···N2^i^	0.85 (1)	2.07 (1)	2.847 (2)	152 (2)
N1—H1n···O5	0.86 (1)	2.09 (1)	2.894 (2)	157 (2)

Symmetry codes: (i) −*x*+1, −*y*+1, −*z*+1.
